# ampliMethProfiler: a pipeline for the analysis of CpG methylation profiles of targeted deep bisulfite sequenced amplicons

**DOI:** 10.1186/s12859-016-1380-3

**Published:** 2016-11-25

**Authors:** Giovanni Scala, Ornella Affinito, Domenico Palumbo, Ermanno Florio, Antonella Monticelli, Gennaro Miele, Lorenzo Chiariotti, Sergio Cocozza

**Affiliations:** 1 Istituto Nazionale di Fisica Nucleare, Sezione di Napoli, Naples, Italy; 2 Dipartimento di Medicina Molecolare e Biotecnologie Mediche, Università degli Studi di Napoli “Federico II”, Naples, Italy; 3 Istituto di Endocrinologia ed Oncologia Sperimentale (IEOS) “Gaetano Salvatore”, Consiglio Nazionale delle Ricerche CNR, Naples, Italy; 4 Dipartimento di Fisica, Università degli Studi di Napoli “Federico II”, Naples, Italy

**Keywords:** DNA methylation, Bisulfite sequencing, Epihaplotype, Epihaplotype based analysis, Methylation profiles

## Abstract

**Background:**

CpG sites in an individual molecule may exist in a binary state (methylated or unmethylated) and each individual DNA molecule, containing a certain number of CpGs, is a combination of these states defining an epihaplotype. Classic quantification based approaches to study DNA methylation are intrinsically unable to fully represent the complexity of the underlying methylation substrate. Epihaplotype based approaches, on the other hand, allow methylation profiles of cell populations to be studied at the single molecule level.

For such investigations, next-generation sequencing techniques can be used, both for quantitative and for epihaplotype analysis. Currently available tools for methylation analysis lack output formats that explicitly report CpG methylation profiles at the single molecule level and that have suited statistical tools for their interpretation.

**Results:**

Here we present ampliMethProfiler, a python-based pipeline for the extraction and statistical epihaplotype analysis of amplicons from targeted deep bisulfite sequencing of multiple DNA regions.

**Conclusions:**

ampliMethProfiler tool provides an easy and user friendly way to extract and analyze the epihaplotype composition of reads from targeted bisulfite sequencing experiments. *ampliMethProfiler* is written in python language and requires a local installation of BLAST and (optionally) QIIME tools. It can be run on Linux and OS X platforms. The software is open source and freely available at http://amplimethprofiler.sourceforge.net.

**Electronic supplementary material:**

The online version of this article (doi:10.1186/s12859-016-1380-3) contains supplementary material, which is available to authorized users.

## Background

Locus-specific DNA methylation analysis is used widely in many research fields. Traditionally, Sanger sequencing was used as the standard technique to quantify the methylation state of a specific bisulfite-treated locus at single nucleotide resolution. Nowadays, next-generation sequencing techniques are used for high-throughput sequencing of bisulfite polymerase chain reaction (PCR) amplicons obtaining many thousands of sequences in a single sequencing run [1, 2]. In such a way, the methylation heterogeneity of a given locus can be studied at the single molecule level.

With high-throughput sequencing of bisulfite PCR amplicons, it is possible to investigate methylation diversity in a sample by looking directly at methylation profiles (epihaplotypes) of the individual cells in a population, rather than considering a single profile where CpG methylation is analyzed as a mixture of methylated and unmethylated CpGs [3]. Analysis of epihaplotype diversity is applicable to fields as diverse as carcinogenesis, developmental biology and plant biology [4–6].

Using this high-throughput approach, the epihaplotypes of the pool of cells that comprise the study sample can be treated as a population of haploid organisms. When considered in this way, notions and techniques derived from other fields, such as population genetics, ecology and metagenomics can be incorporated into protocols. In particular, several metrics, statistical methods and tools developed to analyze population structure can be easily imported and adapted for the analysis of methylation profiles generated from deep targeted sequencing. It is, therefore, important to develop tools that are able to extract locus-specific NGS methylation data in a format that can be easily imported into already available statistical tools, and that allow a user-friendly, basic statistical interpretation of this particular kind of data.

Here, we present ampliMethProfiler, a pipeline for the extraction and analysis of methylation profiles at the single molecule level from deep targeted bisulfite sequencing of multiple DNA regions. This tool provides functions to demultiplex, filter and extract methylation profiles directly from FASTA files. Among the various output formats that are available for the representation of methylation profile composition, *ampliMethProfiler* provides the Biological Observation Matrix (BIOM) [7] format, which allows the user to directly import methylation profiles into a wide range of meta-genomics analysis software tools. Also, several core analyses of the epihaplotype population structure of input samples can be automatically performed by the pipeline using a local installation of QIIME software [8].

## Implementation

### Input data

**Fig. 1:**
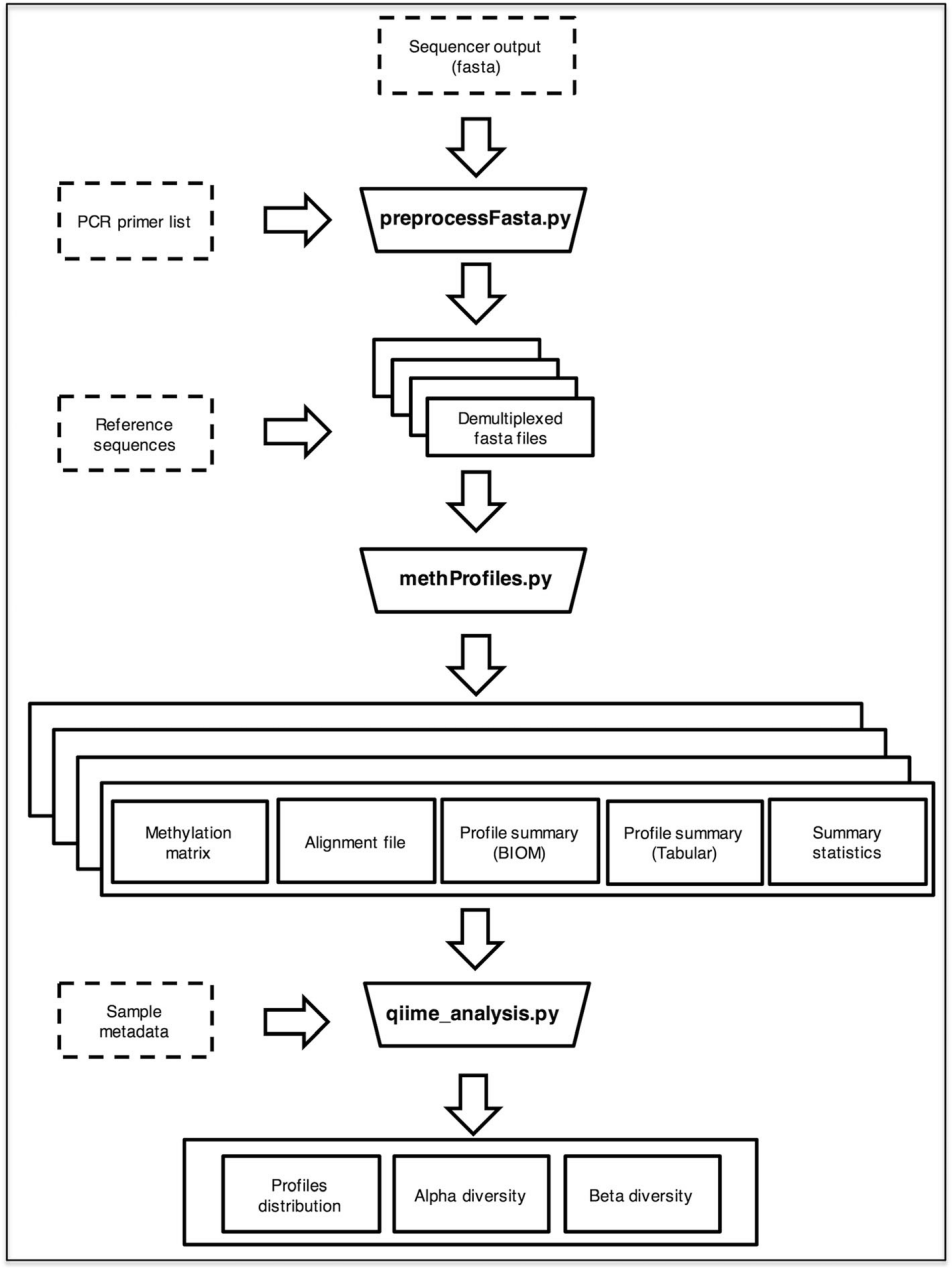
*ampliMethProfiler* workflow. Functional modules are represented as trapezes. Input and output files are represented as dashed and solid rectangles, respectively

*AmpliMethProfiler* (Fig. 1) requires three types of input files: a file containing the reads from the sequencer in FASTA format, a comma-separated file containing information on the sequenced regions, and a FASTA file containing the reference sequences of the analyzed regions. Optionally, a file containing metadata associated with each sample can be provided to enable the tool to perform a series of basic EpiHaplotype based Analyses (EHAs) on the pipeline outcome.

### Demultiplexing and filtering

Reads from targeted bisulfite sequencing of multiple regions are demultiplexed by comparing their 5′ and 3′ ends with a list of provided PCR primers. The demultiplexing procedure is based on a user-provided percentage of similarity between the 5′ or 3′ end of a read sequence and the corresponding PCR primer sequences. Reads are filtered out if no match is found between at least one of the read ends or if, given a user-provided threshold, their length does not match.

### Extraction of methylation profiles

First, amplicons from targeted bisulfite sequencing are aligned to the corresponding bisulfite-converted reference sequence using the locally installed BLASTn program [9]. Then, the tool inspects the C and CpG aligned positions for each input read. Bisulfite efficiency for each aligned read is computed as the percentage of conversion of non-CpG cytosine residues (green Cs in the reference sequence in the example below) to thymine residues (green Ts in the reference and bisulfite-converted reference sequences in the example below). If the percentage of non-CpG deaminated C residues (red Cs in the read sequence in the example below) over the total number of non-CpG C residues is below the given threshold, the read is discarded. In this latter case, positions for which residues other than C or T (A, G) or gaps are found are excluded from the assay (purple characters in the read sequence in the example below). A user provided threshold defines the minimum percentage of reference non-CpG cytosine residues to be assayed to consider the bisulfite efficiency estimate valid; if this percentage is below the given threshold the read is discarded. The methylation profile for each aligned read is determined by evaluating the deamination of CpG sites as a result of the bisulfite treatment.

For each CpG position in the aligned reference sequence (green Cs in the bisulfite-converted reference sequence in the example below), the corresponding position in the aligned read sequence is inspected. If a C is found in that position, then that site is considered methylated; if a T is found, then the site is considered unmethylated; and if alignment gaps or other bases (A or G) are found, the methylation state of the CpG site is reported as uncertain (marked in purple in the example below).

Methylation percentages for each site are then computed as the number of non-deaminated bases mapped on that site over the total number of non-ambiguously mapped positions. The same procedure is used to compute bisulfite efficiency for all C (non-CpG) sites. Then, the abundance of each distinct methylation pattern is evaluated for each sample. Such reports are created by counting, for each of the possible 2^NCpG^ epihaplotypes (where NCpG stands for the number of CpG sites in the analyzed region), the number of passing filter reads containing the pattern.

### EpiHaplotype based analysis

A series of exploratory EHAs are performed on the sample profile abundances obtained in the previous steps. These analyses are performed starting from the BIOM file containing methylation profile abundances and a metadata file reporting the characteristics for each analyzed sample. A local installation of *biom* tool [7] and QIIME software suite are employed for this purpose.

Three kinds of analyses are performed to summarize sample epihaplotype composition:

i) A series of summary statistics on the number of passing filter profiles in each sample are performed using the “*biom summarize-table”* command;
ii) A summary of samples’ taxonomic composition, computed as the abundance of profiles stratified by the number of methylated CpGs, is performed through QIIME’s *summarize_taxa_through_plots.py* module; and
iii) A heatmap, comparing relative abundances of methylation profiles between samples, where profiles (rows) are clustered by UPGMA hierarchical clustering, is created with QIIME’s *make_otu_heatmap.py* script.

Within-sample diversity (Alpha diversity), for samples and groups of samples in the study, is evaluated using QIIME’s *alpha_rarefaction.py* workflow, which performs the following steps:

1. Generate rarefied profile abundance tables for each sample (*multiple_rarefactions.py*);
2. Compute measures of alpha diversity for each rarefied OTU table (*alpha_diversity.py*);
3. Collate alpha diversity results (*collate_alpha.py*); and
4. Generate alpha rarefaction plots (*make_rarefaction_plots.py*).

The between-sample diversity (Beta diversity) between all pairs of samples in the study is evaluated using QIIME’s *beta_diversity_through_plots.py* workflow, which performs the following steps:

1. Rarefy profile abundance tables to remove sampling depth heterogeneity (*single_rarefaction.py*);
2. Compute beta diversity metrics (*beta_diversity.py*) using Bray–Curtis dissimilarity between methylation profile abundances of samples;
3. Run Principal Coordinates Analysis (*principal_coordinates.py*);
4. Generate 3D Emperor PCoA plots (*make_emperor.py*) and 2D PCoA plots (*make_2d_plots.py*); and
5. Compare distances within and between groups of samples using boxplots (*make_distance_boxplots.py*).

## Results

### ampliMethProfiler pipeline

The ***ampliMethProfiler*** pipeline is composed of three functional modules (Fig. 1), implemented in three python modules: preprocessFasta.py, methProfiles.py, qiime_analysis.py. The *preprocessFasta.py* module generates, for each sequenced region, a quality filtered FASTA file containing the reads from that region that passed filtering. Importantly, it creates a new FASTA file for each analyzed region, whose entries are annotated with the ID of the region and of the sample. The *methProfiles.py* module runs on each demultiplexed, filtered FASTA file generated by *preprocessFasta.py* and computes CpG methylation profile matrices, profile counts and several summary and quality statistics. For each analyzed region, *methProfile.py* returns the following output files.

#### Summary and quality statistics file

**Fig. 2:**
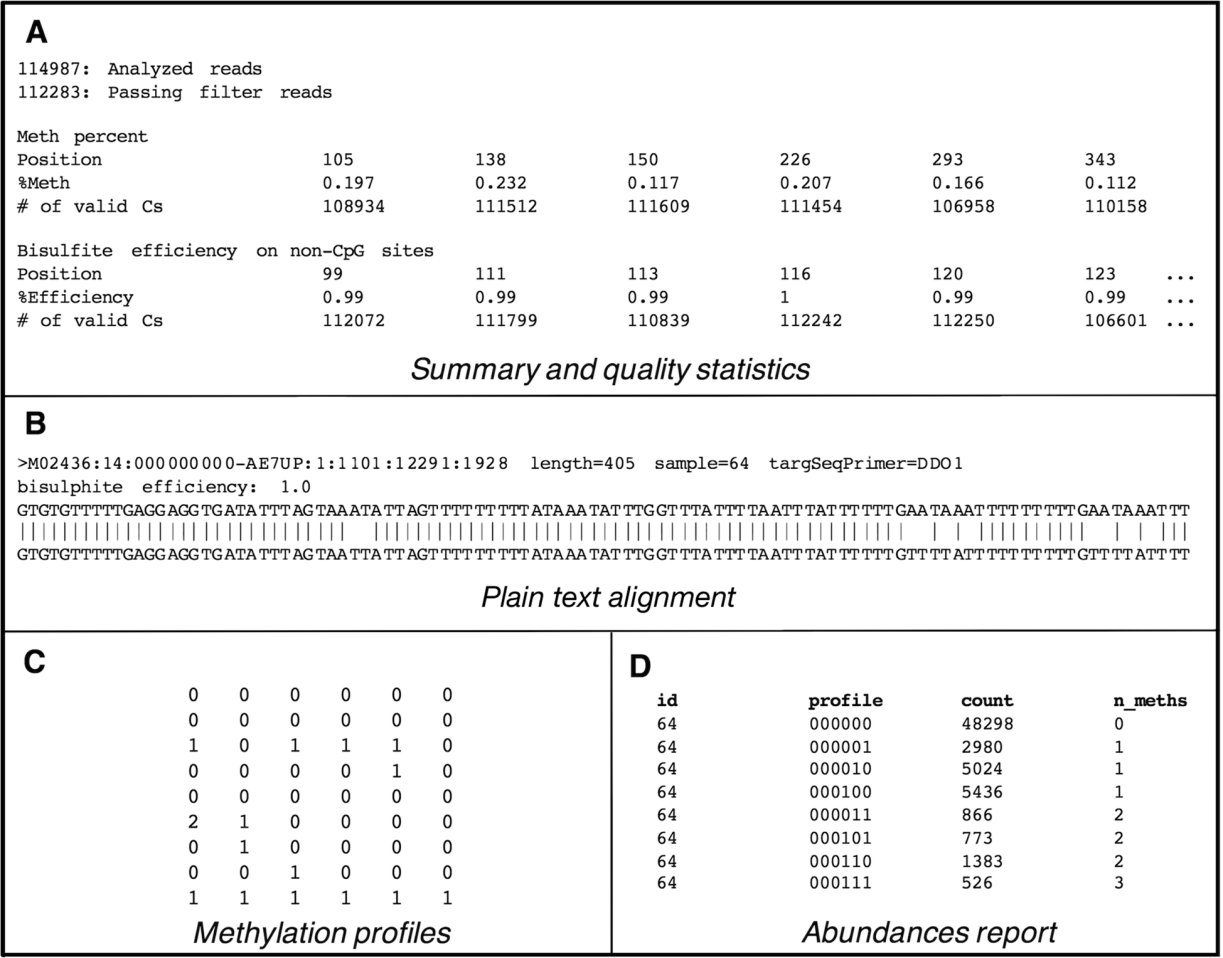
*ampliMethProfiler* output files. **a** Content example of a summary and quality statistics file. **b** Content example of a plain text alignment file. **c** Content example of a methylation profiles file. **d** Content example of a methylation profile abundances file

This file contains information about the number of reads that pass the filtering, the methylation percentage of each C in CpG sites, and the bisulfite efficiency for each C in non-CpG sites (Fig. 2a).

#### Alignment file

These files contain BLAST-aligned sequences in the standard BLAST XML output format and in plain text format. The plain text format (Fig. 2b) allows the user to easily inspect alignments. Each entry of this file contains a filter-passed aligned read, represented by five rows that provide the following information:

- read identification, read length, experiment identification, region identification;
- bisulfite efficiency, calculated as the percentage of deaminated Cs (non-CpG) over all Cs (non-CpG);
- alignment of the read sequence against its bisulfite-converted reference sequence.

#### Methylation profiles file

This file contains the CpG methylation profile matrix (Fig. 2c) in which columns and rows represent CpG sites and single reads, respectively. The methylation status of each CpG site in a read is coded 0 if the site is recognized as unmethylated, 1 if the site is recognized as methylated, and 2 if the methylation state could not be assessed (i.e. because other residues other than C or T or gaps are found). Row entries are reported in the same order as in the “Alignment file”, and column order represents the CpG positions as they appear in the reference sequence. Each row can be considered as the CpG methylation profile of a single read and defines an epihaplotype in subsequent analyses.

#### Profile abundance reports

These files contain counts of the occurrence of each epihaplotype in the sample. Such reports are provided in two formats: tabular and BIOM. Each entry of the tabular file (Fig. 2d) represents a distinct methylation profile along with the following information:

- □*id*: sample identification;
- □*profile*: string representation of the methylation profile;
- □*count*: number of times the profile has been found in the sample; and
- □*n_meths*: number of methylated cytosines characterizing the profile.

The BIOM format is a common general-use format for representing biological samples that uses observation contingency tables. The format is designed for general use in broad areas of comparative-omics and is based on the JSON format [7]. Methylation profile abundances are coded in the rich and sparse BIOM format (version 0.9.1). The methylation profiles are coded as taxonomic units and the number of methylated cytosines constituting each profile, hereafter denoted as methylation class, is used as their first-level grouping factor in an ideal phylogeny. Importantly, BIOM coded files from different samples can be merged together in a single BIOM file using suitable ad-hoc scripts.

Finally, qiime_analysis.py returns a first level of exploratory EHAs on the input sample(s). For each analyzed region a folder is created containing the following reports:

- A text summary file, containing summary statistics about the number of profiles present in the set of input samples. In particular, the file reports the number of samples, the total number of observations (distinct methylation profiles) in all analyzed samples, the total read count, the table density (fraction of epihaplotypes with non-zero frequency), the summary of read counts per sample (min, max, median, mean, standard deviation) and a detailed list of read counts per sample.
- The *profileSummary* folder contains text reports and plots reporting the distribution of methylation classes among samples.
- The file *heatmap.pdf* contains a heatmap representing the distribution of each distinct epihaplotype among all the input samples.
- The *Alpha* folder contains information and plots based on alpha diversity metrics for each provided sample. Five alpha diversity metrics are computed for each sample: number of different methylation profiles in the sample, Shannon entropy, Simpson index, Chao 1 index and number of singletons (number of epihaplotypes characterized by only one occurrence in the sample). Such metrics are computed through a rarefaction procedure to evaluate eventual biases deriving from different sequencing depths.
- The *Beta* folder contains information and plots based on beta diversity between the provided samples. All beta diversity analyses are based on a distance function between samples. To achieve this, Bray–Curtis dissimilarity among the epihaplotype compositions of samples has been employed. The *files bray_curtis_dm.txt* and *bray_curtis_pc.txt* contain pairwise distances among samples and principal component analysis data (eigenvalues, Proportion explained, PCA values for each sample). The *bray_curtis_emperor_pcoa_plot* and the *PCA* folders contain principal coordinate analysis (PCoA) plots in html format. The first plot shows the first three components of the PCoA through an interactive 3D html interface and relies on an EMPEROR browser tool, the second plot shows PCoA plots in 2D using combinations of the first three components. Finally, the *dist_boxplot* folder contains a series of boxplots reporting the distribution of pairwise differences within and between user defined groups of samples.

#### ampliMethProfiler pipeline

The whole set of analyses presented above can be executed by running each module alone on each analyzed sample or runs can be pipelined together. The a*mpliMethProfiler.py* module implements the whole flowchart described above by sequential application (and in parallel when possible). The “Demultiplexing and Filtering” and “Extraction of Methylation Profiles” steps are first applied to each analyzed region and each provided sample separately. Thus, for each region, the module creates a single methylation profile abundance file, in the two formats described above, containing epihaplotype abundances for the whole set of analyzed samples.

Finally, EHAs of each analyzed region are carried out by this module using the BIOM file containing computed abundances for each sample.

## Case study

As a proof of concept, we report *ampliMethProfiler* pipeline analysis of targeted deep bisulfite sequencing of a genomic region in the promoter of the *Ddo* gene from gut tissues of three newborn mice (P0 status) and three adult mice (P90 status).

We analyzed the region spanning from −468 to −63 bp upstream of the transcription start site of the Ddo-201 transcript (40630011 – ENSEMBLE GRCm38.p4 assembly).

To evaluate the methylation levels of the target region, we used a double-step PCR strategy to generate an amplicon library of bisulfite DNA that could be sequenced by an Illumina MiSeq Sequencer.

In the first PCR reaction, we designed primers to generate tiled amplicons. The 5′ ends of these primers contained overhang adapter sequences (Fw: 5′ TCGTCGGCAGCGTCAGATGTGTATAAGAGACAG 3′, Rv: 5′ GTCTCGTGGGCTCGGAGATGTGTATAAGAGACAG 3′) to be used in the second PCR step to add multiplexing indices and Illumina sequencing adapters.

Paired-end reads from Illumina MiSeq sequencing were merged together using the PEAR tool [10] using as threshold a minimum of 40 overlapping residues, then quality filtered using as threshold a mean PHREAD score of at least 33, and finally converted to FASTA format using PRINSEQ [11]. We then used the resulting FASTA files as input to the *ampliMethProfiler* pipeline using the following parameters:

- length ±50% compared with the reference sequence length;
- at least 80% sequence similarity with the primer of the corresponding target region; and
- at least 98% read bisulfite efficiency.

The whole analysis took 23 m 8.45 s on a 2 × 6-core Intel Xeon X5660@2.3 GHz with 64 GB ram, running the Ubuntu 12.04.5 LTS operating system.

Table 1 reports the characterization for each input sample, along with the number of input and passing filter reads.

**Table 1:** Sample characteristics

Mouse	Age	Input reads	Passing filter reads
M1_0	P0	114987	112283
M2_0	P0	48780	48288
M3_0	P0	90636	89114
M4_90	P90	5436	2498
M5_90	P90	28711	27750
M6_90	P90	117228	115069

**Fig. 3:**
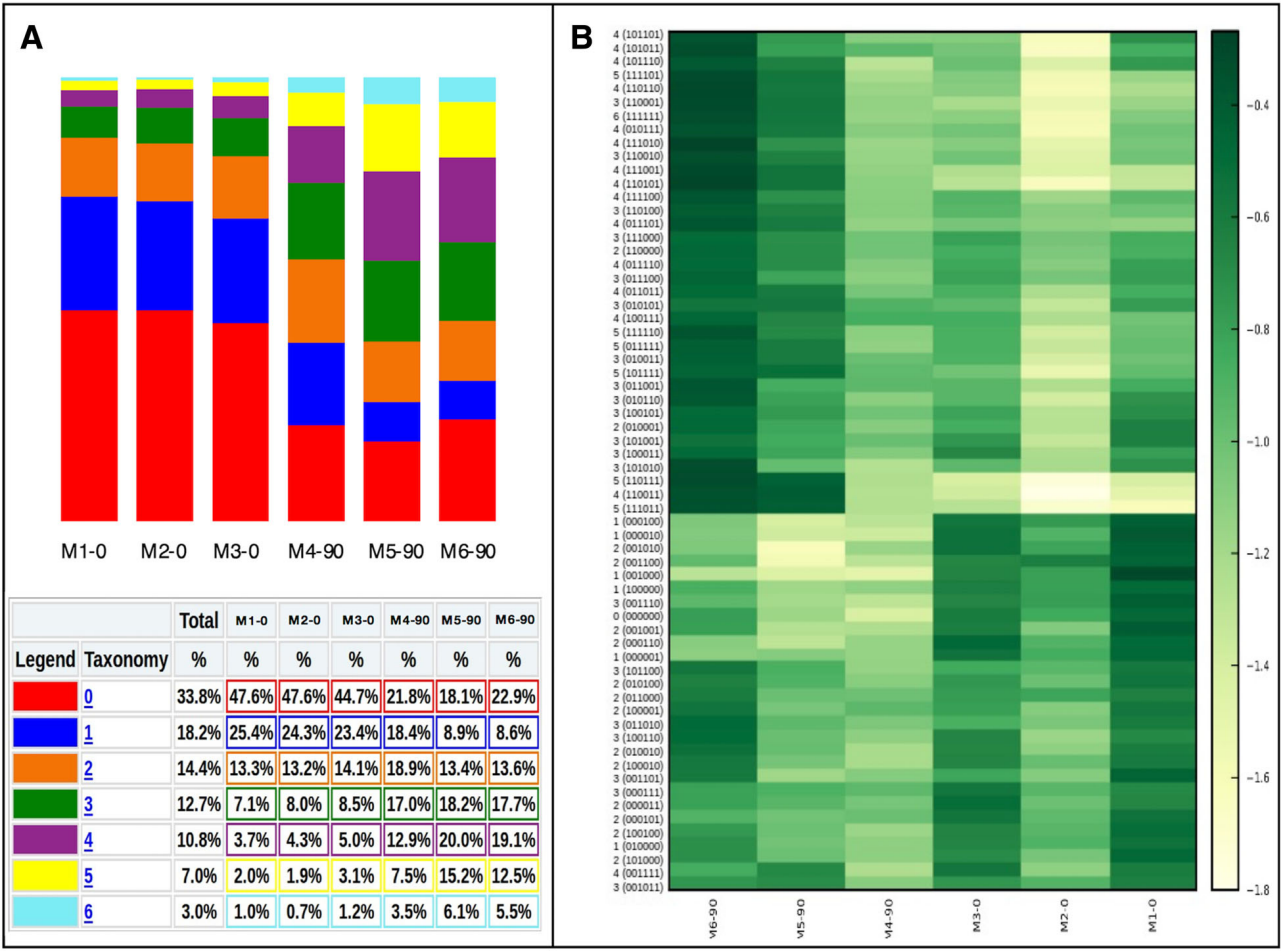
Profile abundances plots. **a** Profile composition summary charts. Bar charts representing relative abundances of profiles grouped by number of methylated CpGs. **b** Heatmap representing methylation profile abundances in each sample

The obtained methylation profile compositions (Additional file 1: Table S1) were then analyzed by the *qiime_analisys.py* module to describe samples by methylation class (Fig. 3a) and epihaplotype frequencies (Fig. 3b). As expected, both analyses showed clear differences between mice at stage P0 and mice at stage P90. The analysis also revealed that profile composition is consistent at a developmental stage in different mice.

**Fig. 4:**
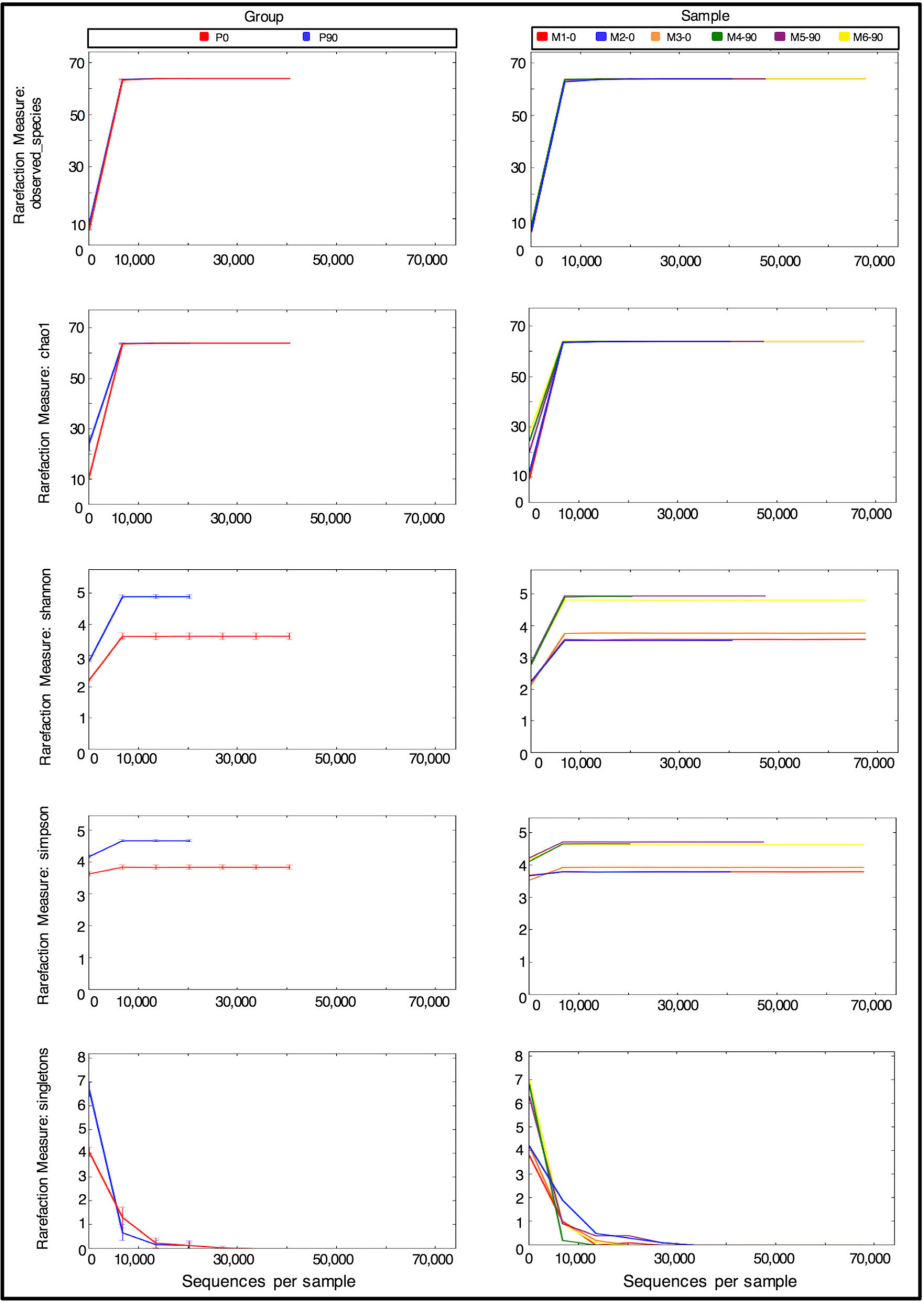
Alpha diversity rarefaction plots at sample level (*right column*) and developmental stage level (*left column*)

Within-sample diversity indices were then computed by the *qiime_analisys.py* module through rarefaction at the minimum depth found in the pool of input samples. Figure 4 shows rarefaction curves computed by *ampliMethProfiler* for five different Alpha diversity metrics: Observed Species, Shannon entropy, Simpson index, Chao 1 index and number of singletons (profiles which appear only once in the sample). Alpha diversity curves are provided for each sample, as well as averages for the two groups along with the corresponding confidence intervals.

Also in this case, the analysis was able to identify differences between the two groups of mice, and in particular which phenotype (newborn vs. adult) was richer in terms of epihaplotype composition. In this case, P0 mice showed a more heterogeneous composition than fully developed mice. Finally, between-sample diversity was computed for the two groups of samples. We let the tool compute distances between epihaplotype composition of input samples using Bray-Curtis distance.

**Fig. 5:**
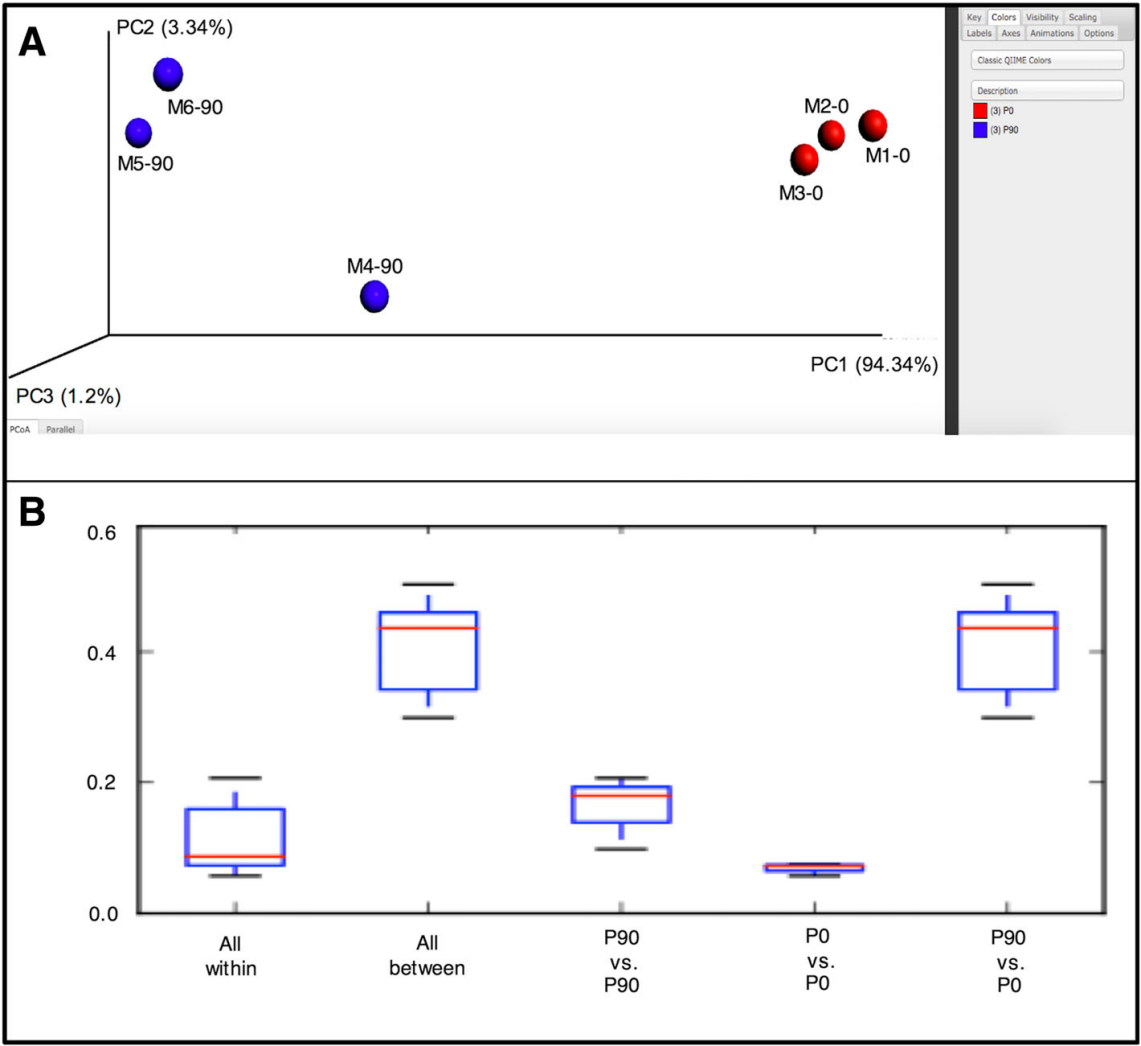
Beta diversity plots. **a** 3D Emperor plot snapshot representing the first three principal components of the PCoA. **b** From left to right are reported: Bray-Curtis distance boxplots of pairwise distances computed between samples from the same developmental stage, pairwise distances computed between pairs of samples from different developmental stages, distances within P90 mice, distances within P0 mice and distances between pairs of P90 and P0 mice

Differences in epihaplotype composition between the two developmental stages are represented by means of PCoA plots. Figure 5a reports the layout of a 3D Emperor plot of the first three principal components from PCoA with colors representing the two developmental stages. Samples from the two groups clearly separate in the 3D space and also tend to cluster together.

Distance boxplots of epihaplotype composition are a useful graphical tool to validate this last statement. In particular, the *qiime_analisys.py* module analyzes and summarizes distances within and between user defined groups of samples, reporting distributions of distances through a series of boxplots.

The first two boxplots of Fig. 5b show how distances between pairs of samples from the same group are appreciably lower than distances between pairs from different groups. Finally, the third and fourth boxplots show that methylation profiles of P0 samples are on average 2-fold closer to each other compared with those of P90 samples.

## Discussion

In this manuscript we present the *ampliMethProfiler* pipeline, a tool aimed at EpiHaplotype based analysis of data from targeted deep bisulfite sequencing experiments.

Classic quantitative methylation analyses only consider percentages of methylation by characterizing each CpG site in a region, thus flattening the information on local conformation heterogeneity carried in the pool of analyzed amplicons. These kinds of approaches unavoidably mask the intrinsic complexity of the local methylation patterns in each cell of an analyzed sample. Epihaplotype based approaches, on the other hand, offer the possibility to study the methylation state of a sample from a complementary point of view, namely by considering the methylation conformation of each single molecule in the pool of analyzed cells.

To perform such analyses with sufficient power for a biological sample, it is essential to analyze the methylation profiles of a very large number of sequences. This can now be accomplished through targeted deep sequencing of bisulfite-treated DNA.

Analyzing the methylation conformation of single reads in a multi-clonal population, such as cells from cancer tissues, offers the possibility to track the progression of distinct methylation patterns among different pathological forms/stages. A similar approach has been adopted in the study of driver and passenger DNA mutations in various form of cancer [12]. Likewise, the proposed epihaplotype based approach to study methylation patterns, if applied at relevant selected genomic regions, such as promoters of cancer-related genes, could lead to the discovery of driver and passenger epi-mutations.

The approach proposed here is based on the idea that the epihaplotype composition of a sample can be considered as a biological community, were each distinct methylation profile can be studied exactly as a distinct taxonomical unit is studied in a metagenomics analysis. In this way, several notions and metrics used in ecology and population genetics can be exploited to describe the heterogeneous methylation patterns in a population of cells from a sample and to assess the compositional differences between different samples. For example, the diversity and distribution of methylation profiles characterizing a sample can be described with Alpha diversity metrics, such as the number of different taxonomic units or the Shannon entropy index. Likewise, differences among epihaplotype compositions of samples can be measured through Beta diversity metrics, such as Bray-Curtis distance or Euclidean distance.

The recent diffusion of metagenomics analyses, linked to the advent of microbiome analysis from raw DNA sequencing data, was accompanied by the production of multiple bioinformatics tools for the analysis of biological communities [13], as well as the development of standards to represent biological communities. One of the most widely used formats in this field is the BIOM, which is recognized by the vast majority of tools for the analysis of biological communities. In this regard, it can be useful to represent epihaplotype compositions as biological observation matrices. In fact, this format gives the possibility to carry out EHAs on methylation data by taking advantage of the already available repository of tools available for ecology and metagenomics.

The *ampliMethProfiler* tool provides a complete analysis pipeline that, starting from FASTA files containing reads from targeted bisulfite sequencing experiments, extracts methylation profiles from the input samples along with a series of exploratory analyses of their profile compositions. It provides functions to demultiplex, filter and quality check input reads along with the classic quantitative assessment of CpG methylation percent per site.

By taking advantage of the local installation of the QIIME suite, *ampliMethProfiler* enables a series of basic exploratory analyses of the methylation profiles in the given experimental samples. The core set of the analyses provided by *ampliMethProfiler* were chosen to be instrumental for all studies investigating methylation patterns. If more specific analyses are needed, the BIOM files produced by the tool, in combination with the vast collection of QIIME scripts, enable the user to easily perform more sophisticated tasks depending on the specific experimental design.

Table 2 presents a comparison of *ampliMethProfiler* with state of the art tools for methylation analysis of bisulfite sequencing experiments. In particular, several tools have been described in the literature for the analysis of bisulfite sequencing data [14] but the majority of them were designed to explicitly provide quantitative measurement of methylation for each analyzed CpG site. Few of these tools provide outputs containing a direct representation of methylation profiles for each analyzed read and none provide output formats and statistical tools that are specifically designed for EHA of methylation heterogeneity.

**Table 2:** Comparison of existing software programs for bisulfite sequencing analysis (Adapted from [14])

Software	Programming Language and Implementation	Analysis Process	Visual Output	Input File	Output File	EHA	Epihaplotype Counts	Experiment Quality Check
MethPat	Python, pip install, URL available to install files locally.	Summarises Bismark output.	Interactive HTML and summary text file of epihaplotype counts. Scalable PNG file.	Bismark methylation extractor output, user-defined BED format file.	HTML and tab delimited text file.	No	Yes	No, made by Bismark.
Bismark	Command line, Python, requires bwa.	Performs alignment to bisulfite reference genome.	None, generates BAM files for visualisation with SeqMonk or IGV.	FASTQ file.	BAM and tab delimited text files.	No	No	Yes computes C to T conversion.
BSPAT	Java/JSP web interface.	Visualization and summarization of Bismark output.	PNG file and UCSC Genome Browser file.	Bismark output, FASTQ files.	Text file summary, PNG and UCSC Genome Browser BED file.	No	Yes	No
MPFE	R library, Bioconductor.	Calculates probabilities that epihaplotypes are true.	R image outputs.	Table of read counts from bisulfite sequencing data.	Derived statistics and plots.	No	Yes	Yes
Methylation plotter	R library, shiny interactive web application.	Visualizes beta DNA methylation values.	Interactive webpage with setting options to adjust a static image of DNA methylation values for each sample. PNG and PDF output.	Text file containing matrix of sample vs beta value at each CpG of interest.	PDF and PNG image file.	No	No	No
RnBeads	R library, Bioconductor.	Processes summary data from other software for visualization.	Interactive HTML and UCSC Genome browser track hub files. PNG files.	BED file	HTML summary	No	No	Yes
coMET	R library, Webserver for analysis.	For EWAS studies. Analyses derived matrix files.	Image files of plots with genomic locations.	Text matrix files	Image files	No	No	No
AmpliMethProfiler	Python, BLAST and QIIME	Filtering and de-multiplexing of the sequence, generation of the methylation status and EpiHaplotype composition analysis.	HTML plots and summary text file. An heatmap in PDF format. An Alpha and a Beta diversity plot in HTML and PDF format.	A fasta directory with all fasta for each sample. A file containing the reads from the sequencer. A metaFile containing information about the samples.	Filtered Fasta file. Blast aligned sequences in XML and TXT format. Summary and quality statistics for region. CpG methylation profile matrix. BIOM file with number of occurrences.	Yes	Yes	Yes, quality statistic for each analyzed region.

The computation and listing of epihaplotype abundances are certainly important, but, especially when the number of samples (and groups) begins to grow, it’s essential to provide biologists with statistical tools able to quantify and summarize the individual sample composition and the differences between samples.

Compared to existing tools, ampliMethProfiler pipeline offers two main advantages:

1. It automatically provides a large number of statistical analyses and representations of intra- and inter-sample diversity in term of their epihaplotype composition;
2. It provides epihaplotype abundances in several output formats, which, in turn, are easy to import in other statistical and/or population genetics tools that are borrowed from ecology.

## Conclusions

In conclusion, our tool provides an easy and user friendly way to extract and analyze the epihaplotype composition of reads from targeted bisulfite sequencing experiments. *ampliMethProfiler* is written in python language and requires a local installation of BLAST and (optionally) QIIME tools. It can be run on Linux and OS X platforms. The software is open source and freely available at http://amplimethprofiler.sourceforge.net.

## Availability of data and materials

Project name: AmpliMethProfiler

Project home page: https://sourceforge.net/projects/amplimethprofiler.

Operating system(s): Linux, MacOS X.

Programming language: Python.

Other requirements: Biom 2.1.5 or higher (optional), QIIME 1.9 or higher (optional), Biopython 1.65 or higher, Blast 2.2.25 or higher (suggested).

License: GNU GPL.

## Additional file

Additional file 1: Table S1. The table reports for each possible epihaplotype the number of methylated CpG and the number of filter-passed aligned reads containing the epihaplotype in each sample. (DOCX 100 kb)

## Abbreviations

BIOM: Biological observation matrix
EHA: EpiHaplotype based analysis
NCpG: Number of CpG sites in a region
NGS: Next generation sequencing
OTU: Operational taxonomic unit
PCA: Principal components analysis
PCoA: Principal coordinate analysis
PCR: Polymerase chain reaction
