# Supplementary material for: ampliMethProfiler: a pipeline for the analysis of CpG methylation profiles of targeted deep bisulfite sequenced amplicons
Source: BMC Bioinformatics. 2016 Nov 25;17:484. doi: 10.1186/s12859-016-1380-3 (PMC5123276; doi:10.1186/s12859-016-1380-3)
Supplement: Additional file 1: Table S1. — The table reports for each possible epihaplotype the number of methylated CpG and the number of filter-passed aligned reads containing the epihaplotype in each sample. (DOCX 100 kb) [file 12859_2016_1380_MOESM1_ESM.docx]

| profile | n_meths | M1-0 | M2-0 | M3-0 | M4-90 | M5-90 | M6-90 |
| --- | --- | --- | --- | --- | --- | --- | --- |
| 000000 | 0 | 48298 | 20969 | 36177 | 5493 | 9764 | 23722 |
| 000001 | 1 | 2980 | 1220 | 2576 | 628 | 720 | 676 |
| 000010 | 1 | 5024 | 1515 | 3649 | 525 | 522 | 1040 |
| 000100 | 1 | 5436 | 2498 | 3948 | 743 | 557 | 1273 |
| 001000 | 1 | 1523 | 486 | 681 | 98 | 109 | 158 |
| 010000 | 1 | 5450 | 2417 | 4197 | 1450 | 1825 | 3645 |
| 100000 | 1 | 5300 | 2565 | 3849 | 1197 | 1057 | 2128 |
| 000011 | 2 | 866 | 406 | 1248 | 369 | 478 | 628 |
| 000101 | 2 | 773 | 297 | 730 | 274 | 255 | 331 |
| 000110 | 2 | 1383 | 519 | 1419 | 300 | 208 | 337 |
| 001001 | 2 | 175 | 37 | 105 | 25 | 24 | 70 |
| 001010 | 2 | 154 | 63 | 122 | 29 | 10 | 36 |
| 001100 | 2 | 372 | 253 | 225 | 54 | 28 | 116 |
| 010001 | 2 | 680 | 154 | 396 | 230 | 427 | 967 |
| 010010 | 2 | 1075 | 336 | 957 | 273 | 466 | 1330 |
| 010100 | 2 | 1866 | 674 | 1178 | 531 | 846 | 1538 |
| 011000 | 2 | 1005 | 659 | 728 | 435 | 442 | 1079 |
| 100001 | 2 | 474 | 145 | 285 | 202 | 162 | 497 |
| 100010 | 2 | 878 | 359 | 755 | 211 | 324 | 903 |
| 100100 | 2 | 1236 | 469 | 884 | 271 | 396 | 692 |
| 101000 | 2 | 447 | 124 | 273 | 103 | 135 | 259 |
| 110000 | 2 | 2068 | 1338 | 2114 | 1456 | 3030 | 5311 |
| 000111 | 3 | 526 | 301 | 686 | 282 | 324 | 393 |
| 001011 | 3 | 45 | 23 | 34 | 15 | 38 | 34 |
| 001101 | 3 | 119 | 27 | 50 | 27 | 22 | 86 |
| 001110 | 3 | 154 | 65 | 86 | 20 | 25 | 45 |
| 010011 | 3 | 213 | 84 | 275 | 223 | 546 | 795 |
| 010101 | 3 | 290 | 82 | 194 | 216 | 460 | 474 |
| 010110 | 3 | 689 | 149 | 436 | 290 | 574 | 1543 |
| 011001 | 3 | 115 | 47 | 98 | 94 | 113 | 351 |
| 011010 | 3 | 323 | 116 | 170 | 114 | 144 | 417 |
| 011100 | 3 | 686 | 388 | 649 | 322 | 633 | 1481 |
| 100011 | 3 | 201 | 73 | 260 | 102 | 188 | 400 |
| 100101 | 3 | 198 | 53 | 155 | 97 | 172 | 327 |
| 100110 | 3 | 382 | 126 | 407 | 140 | 189 | 587 |
| 101001 | 3 | 55 | 11 | 42 | 24 | 33 | 67 |
| 101010 | 3 | 80 | 26 | 48 | 23 | 45 | 198 |
| 101100 | 3 | 236 | 105 | 127 | 65 | 119 | 239 |
| 110001 | 3 | 255 | 107 | 224 | 270 | 1002 | 1793 |
| 110010 | 3 | 594 | 216 | 599 | 437 | 1443 | 2881 |
| 110100 | 3 | 863 | 720 | 1047 | 708 | 2073 | 3489 |
| 111000 | 3 | 1160 | 791 | 1319 | 822 | 1648 | 2705 |
| 001111 | 4 | 84 | 26 | 92 | 20 | 72 | 48 |
| 010111 | 4 | 254 | 65 | 251 | 211 | 686 | 1151 |
| 011011 | 4 | 105 | 45 | 101 | 69 | 194 | 243 |
| 011101 | 4 | 145 | 154 | 185 | 147 | 518 | 852 |
| 011110 | 4 | 342 | 170 | 314 | 175 | 425 | 683 |
| 100111 | 4 | 105 | 64 | 153 | 153 | 244 | 381 |
| 101011 | 4 | 25 | 4 | 17 | 21 | 29 | 85 |
| 101101 | 4 | 72 | 9 | 31 | 30 | 64 | 165 |
| 101110 | 4 | 103 | 21 | 60 | 30 | 135 | 236 |
| 110011 | 4 | 144 | 70 | 188 | 242 | 1768 | 2009 |
| 110101 | 4 | 228 | 114 | 251 | 374 | 1366 | 2500 |
| 110110 | 4 | 398 | 168 | 539 | 464 | 1763 | 3310 |
| 111001 | 4 | 120 | 87 | 141 | 182 | 701 | 1227 |
| 111010 | 4 | 329 | 114 | 294 | 244 | 671 | 1924 |
| 111100 | 4 | 1279 | 763 | 1392 | 904 | 2155 | 4909 |
| 011111 | 5 | 306 | 135 | 382 | 214 | 728 | 1109 |
| 101111 | 5 | 61 | 17 | 54 | 63 | 171 | 199 |
| 110111 | 5 | 279 | 130 | 277 | 410 | 2599 | 3494 |
| 111011 | 5 | 73 | 60 | 162 | 169 | 1168 | 1393 |
| 111101 | 5 | 455 | 177 | 557 | 404 | 1784 | 3184 |
| 111110 | 5 | 837 | 337 | 1077 | 637 | 1719 | 3549 |
| 111111 | 6 | 1021 | 314 | 942 | 872 | 3296 | 5735 |
